# Isoalantolactone Enhances the Antitumor Activity of Doxorubicin by Inducing Reactive Oxygen Species and DNA Damage

**DOI:** 10.3389/fonc.2022.813854

**Published:** 2022-01-25

**Authors:** Fengjiao Wu, Rongrong Shao, Peisen Zheng, Tingting Zhang, Chenyu Qiu, Hehuan Sui, Shaotang Li, Libo Jin, Huanle Pan, Xiance Jin, Peng Zou, Ri Cui, Congying Xie

**Affiliations:** ^1^ The First Affiliated Hospital of Wenzhou Medical University, Wenzhou Medical University, Wenzhou, China; ^2^ Cancer and Anticancer Drug Research Center, School of Pharmaceutical Sciences, Wenzhou Medical University, Wenzhou, China; ^3^ Wenzhou University-Wenzhou Medical University Collaborative Innovation Center of Biomedical, Wenzhou, China; ^4^ Biomedical Collaborative Innovation Center of Zhejiang Province, Wenzhou University, Wenzhou, China

**Keywords:** colon cancer, ROS, isoalantolactone, JNK, doxorubicin, DNA damage

## Abstract

Colon cancer is one of the most common cancer in the world. Doxorubicin (DOX) is a classical anti-tumor drug which widely used in treatment of cancers, however, high toxicity limited its further clinical application. Thus, it is urgent to find new drugs with low toxicity and high efficiency to treat colon cancer. Isoalantolactone (IATL), an isomeric sesquiterpene lactone isolated from the plant of inula helenium, has been reported to have anti-cancer activity against a variety of cancer cells. However, the function of IATL in colon cancer remains unclear. Here, we demonstrated that IATL inhibited colon cancer cell growth by increasing cellular reactive oxygen species (ROS) production. Further study showed that ROS accumulation contributed to DNA damage and JNK signaling pathway activation. In addition, we found that IATL markedly enhanced DOX-induced cell cytotoxicity in colon cancer cells. IATL in combination with DOX significantly increased the ROS production, induced DNA damage and activated JNK signaling pathway. Taken together, our data suggested that combined treatment with IATL and DOX may serve as a potential therapeutics for colon cancer.

## Introduction

Colon cancer is one of the most common cancer with high morbidity and mortality in the world (1). Currently, surgery, chemotherapy and radiotherapy are main therapeutic methods for colon cancer. Although surgical treatment is effective in the early stage of colon cancer, chemotherapy is still a key approach used for colon cancer therapy (2). Doxorubicin is one of the most classical chemotherapeutics and has been extensively used for cancer therapy (3, 4). However, drug resistance and serious side effects limited its further clinical application (5, 6). Therefore, developing new therapeutic drugs that can effectively treat colon cancer is urgent and essential.

Accumulating evidences have suggested that a variety of natural products have a long history in the treatment of cancer (7–9). Isoalantolactone (IATL) isolated from the plant of inula helenium exhibited a wide range of biological activities such as anticancer, antioxidant and anti-inflammation (10–12). Previous studies have reported that IATL had a highly selective cytotoxic effect for cancer cells, but had a low toxicity to human normal cells (13). The specific inhibitory effect of IATL on lung, breast and pancreatic cancers has also been demonstrated (10, 13, 14). However, the role of IATL in colon cancer remains unclear, and the underlying mechanisms are still largely unknown.

Extensive reports have shown that ROS are closely associated with cancers. The level of ROS in various types of tumor cells is higher than that in normal cells, but exorbitant ROS can mediate cancer cells to die (15, 16). Thus, compounds that targeting ROS metabolism can selectively kill cancer cells by elevating the level of ROS above the toxicity threshold. Controlling the level of ROS is a promising strategy to selectively kill cancer cells (17–19). In the present study, we evaluated the effect of IATL on the viability of two different colon cancer cell lines, and investigated the molecular mechanisms underlying the action of IATL.

## Materials and Methods

### Chemicals and Antibodies

Isoalantolactone (IATL) was obtained from Chengdu Herbpurify (Chengdu, China). JNK inhibitor SP600125 was purchased from TargetMol (Boston, USA). Doxorubicin (DOX) and N-Acetyl-L-cysteine (NAC) were purchased from Aladdin Industrial Corporation (Shanghai, China). Antibodies of p-JNK, JNK and cleaved-caspase-3 were purchased from Cell Signaling Technology (Danvers, USA). The 53BP1 antibody was provided from Novus Biologicals (Littleton, CO, USA).

### Cell Lines and Cell Culture

HCT116 cells were propagated in McCoy’s 5A medium supplemented with 10% fetal bovine serum (FBS). HCT-15 cells were maintained in RPMI-1640 medium with 10% FBS. NRK-52E cells were maintained in DMEM plus 10% FBS. MIHA cells were maintained in RPMI-1640 medium with 10% FBS. Cells were propagated in a humidified atmosphere with 5% CO_2_ at 37°C.

### Cell Viability Assay

Approximately 2×10^5^ cells per well were seeded in 6-well plates and allowed to adhere overnight in the incubator at 37°C. The cells were then treated with different concentrations of IATL or DOX for 24 h, cell viability was determined by trypan blue exclusion. The percentage of viable cells relative to DMSO-treated cells is indicated. Combination index (CI) values were calculated using the Chou-Talalay method (20).

### Assessment of ROS Production

Intracellular ROS production was detected by fluorescent probe 2,7-dichlorofluorescein diacetate (DCFH-DA). HCT116 and HCT-15 cells were propagated in 6-well plates. The cells were then treated with IATL or DOX alone or their combination for the indicated times. Subsequently, DCFH-DA was added to the 6-well plates for 30 min before collecting. For quantitative assessment of intracellular ROS levels, green fluorescence was detected and analyzed by fluorescence microscope.

### Immunofluorescence Staining

HCT116 and HCT-15 cells were seeded on sterile cover glasses placed in the 6-well plates, incubated overnight and then treated with IATL or DOX alone or their combination for 20 h. For immunofluorescence, the cells were incubated with primary antibody (53BP1, 1:2,000 dilution) overnight at 4°C, followed by secondary antibodies for 90 min at room temperature. HCT116 and HCT-15 cells were stained with 4′,6-diamidino-2-phenylindole dihydrochloride (DAPI) at room temperature for 10 min. The images were captured with fluorescence microscope.

### Western Blotting Analysis

HCT116 and HCT-15 cells were treated with the test compounds. Cells were washed once with 1 ml of PBS and lysed using cold cell lysis buffer. After centrifuged, soluble fractions were mixed with 5×loading buffer and heated at 100°C for 10 min. The same amount of cell lysates were solubilized in SDS-polyacrylamide gel electrophoresis (PAGE). The separated proteins were transferred to PVDF membranes. Membranes were blocked at room temperature for 1.5 h in 5% non-fat milk plus TBST, and then incubated with primary antibodies overnight at 4°C. HRP-conjugated secondary antibodies and ECL substrate were used for detection.

### Statistical Analyses

Data were analyzed by using GraphPad Prism 5.0 software. Student’s t-test analysis was used to determine the significance of the differences. A probability (P) value of 0.05 or less was considered statistically significant.

## Results

### IATL Elevates ROS Levels and DNA Damage in Colon Cancer Cells

**Figure 1:**
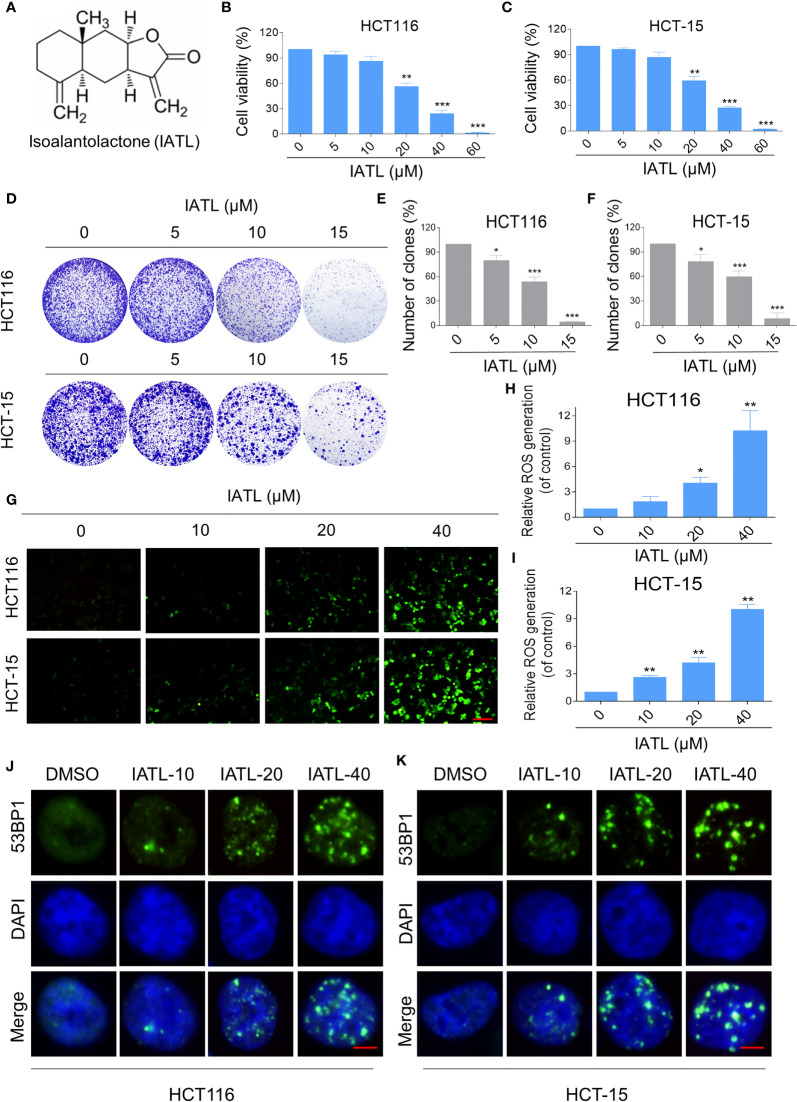
IATL inhibits the cell growth and increases ROS levels in colon cancer cells. **(A)** The chemical structure of IATL. **(B, C)** HCT116 and HCT-15 cells were treated with indicated doses of IATL for 24 h, the cell viability was measured by trypan blue exclusion. **(D–F)** HCT116 and HCT-15 cells were seeded into 6-well plates and then treated with various dosages of IATL as indicated. The number of colonies were assessed and quantified by crystal violet staining. **(G–I)** Intracellular ROS levels were measured in HCT116 and HCT-15 cells after treated with various dosages of IATL for 1 h. Scale bar = 100 µm. **(J, K)** The nuclear foci formation of 53BPl was detected after treated with various dosages of IATL for 20 h in HCT116 and HCT-15 cells. Scale bar = 5 µm. Data from three technical replicates (*p < 0.05, **p < 0.01, ***p < 0.001).

To determine the cytotoxic effect of IATL (**Figure 1A**) in human colon cancer cells, HCT116 and HCT-15 cells were treated with the different concentrations (5, 10, 20, 40, 60 μM) of IATL for 24 h. The results indicated that IATL treatment decreased the viability of HCT116 and HCT-15 cells in a dose-dependent manner (**Figures 1B, C**). Furthermore, we found that IATL treatment significantly suppressed the number of colony formation (**Figures 1D–F**), suggesting that IATL treatment inhibited the colony forming ability of colon cancer cells.

At present, a large number of studies have shown that ROS accumulation in different cancer cells caused by some natural products is one of the mechanisms underlying their cytotoxicity (17, 18). To demonstrate whether ROS was involved in the cell death, we measured the intracellular ROS levels after IATL treatment. The results indicated that IATL promoted ROS accumulation in a dose-dependent manner (**Figures 1G–I**). Superfluous ROS production can cause various cellular damages including oxidative damage to lipids and DNA. Using an immunofluorescence assay, we observed that the accumulation of nuclear 53BP1 foci in HCT116 and HCT-15 cells (**Figures 1J, K**). These data suggest that IATL treatment significantly increased DNA damage in colon cancer cells.

### ROS Accumulation Plays a Critical Role in the IATL-Induced Cytotoxicity in Colon Cancer Cells

**Figure 2:**
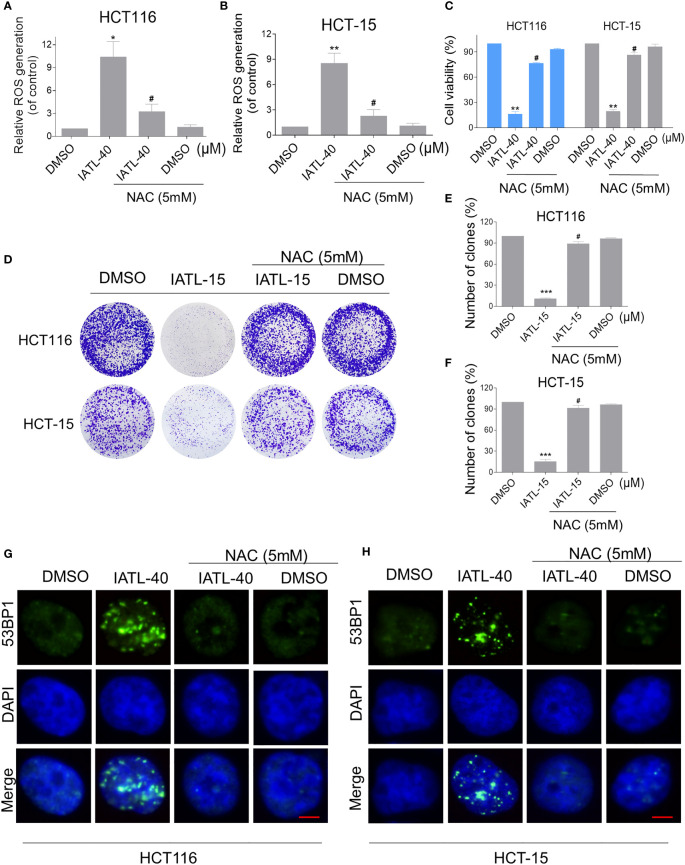
ROS accumulation plays a critical role in the IATL-induced cytotoxicity in colon cancer cells. **(A, B)** Cells were pretreated with NAC (5 mM) for 1 h before exposure to IATL. Intracellular ROS levels were measured after treated with IATL (40 µM) for 1 h. **(C)** Cells were pretreated with NAC (5 mM) for 1 h and cell viability was measured after treated with IATL for 24 h. **(D–F)** Cells were pretreated with NAC (5 mM) for 1 h and then treated with IATL (40 µM). The number of colonies were assessed and quantified by crystal violet staining. **(G, H)** Cells were pretreated with NAC (5 mM) for 1 h and then treated with IATL (40 µM) for 20 h, the nuclear foci formation of 53BPl was detected. Scale bar = 5 µm. Data from three technical replicates (*p < 0.05, **p < 0.01, ***p < 0.001 vs DMSO; ^#^p < 0.05 vs IATL-40 or IATL-15).

To explore the relationship between ROS production and IATL-induced cell death, we used a classical antioxidant, NAC, to scavenge ROS and determine the effects on these cells. Interestingly, we observed that pretreatment with NAC significantly attenuated the IATL-induced cell death in HCT116 and HCT-15 cells (**Figures 2A–C**). In terms of cell function assays, NAC pretreatment greatly attenuated the inhibition of IATL-induced colony formation in two colon cancer cell lines (**Figures 2D–F**). In addition, we tested the interaction between DNA damage and ROS generation. We found that IATL-induced accumulation of nuclear 53BP1 foci were markedly reversed by NAC pretreatment in HCT116 and HCT-15 cells (**Figures 2G, H**). These findings indicated that ROS generation plays a pivotal role in the IATL-induced cytotoxicity in colon cancer cells.

### IATL and DOX Cooperated to Induce ROS-Dependent Cell Death in Colon Cancer Cells

**Figure 3:**
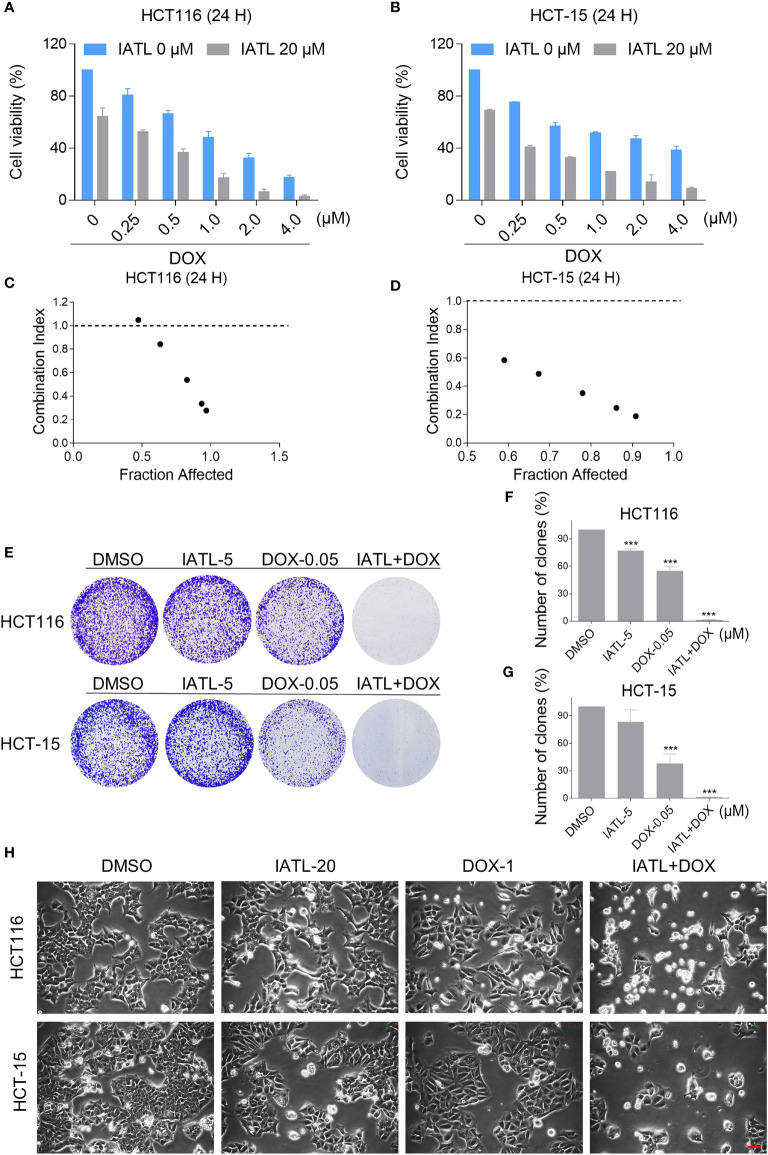
IATL increases the cytotoxicity of DOX in HCT116 and HCT-15 cells. **(A, B)** Cell viability was measured in HCT116 and HCT-15 cells after treated with IATL or DOX alone or their combination for 24 h. **(C, D)** Combination index (CI) values were calculated using the Chou-Talalay method. **(E–G)** The number of colonies were assessed after treated with IATL or DOX alone or their combination. **(H)** Cell morphology was observed after treated with IATL or DOX alone or their combination. Scale bar = 25 µm. Data from three technical replicates (***p < 0.001).

To define whether IATL can synergize with DOX to kill cancer cells, we first tested the effect of IATL or DOX alone or their cotreatment on the viability of HCT116 and HCT-15 cells. In our study, we selected the medium concentration of IATL (20 μM) for combination experiment. We observed that 20 μM IATL markedly increased the cytotoxicity of DOX in HCT116 and HCT-15 cells (**Figures 3A, B**). The combination index (CI) values suggested that IATL in combination with DOX exhibited a synergistic effect against both HCT116 and HCT-15 cells (**Figures 3C, D**). In addition, we found that the combined treatment significantly decreased the colony formation and cell proliferation (**Figures 3E–H**). By contrast, IATL did not markedly increase the cytotoxicity of DOX, and the combined treatment has little effect on normal MIHA and NRK-52E cells (**Figures S1A, B**). Together, these data demonstrate that IATL and DOX exhibit a synergistic effect against human colon cancer cells.

**Figure 4:**
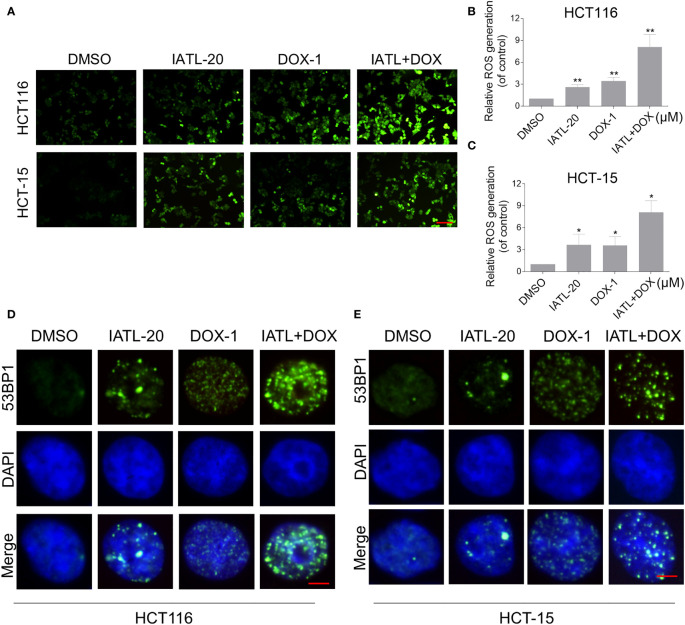
The combined treatment markedly increased ROS levels in HCT116 and HCT-15 cells. **(A–C)** Intracellular ROS levels were measured in HCT116 and HCT-15 cells after treated with IATL or DOX alone or their combination for 1 h. Scale bar = 100 µm. **(D, E)** The nuclear foci formation of 53BPl was detected after treated IATL or DOX alone or their combination for 20 h in HCT116 and HCT-15. Scale bar = 5 µm. Data from three technical replicates (*p < 0.05, **p < 0.01).

Then, we want to investigate the potential synergy mechanisms of IATL and DOX. Previously, we have indicated that IATL treatment increased ROS accumulation in colon cancer cells, which may be the basis of its anticancer activity. As shown in **Figures 4A–C**, compared with IATL or DOX treatment alone, the combined treatment markedly increased ROS levels in HCT116 and HCT-15 cells. Moreover, using an immunofluorescence assay, we observed that the combined treatment increased the accumulation of nuclear 53BP1 foci (**Figures 4D, E**). The cleaved-caspase-3 is considered to be a reliable marker for apoptotic cell death. Therefore, we detected the expression of cleaved-caspase-3 in HCT116 cells. We found that IATL and DOX combination treatment significantly increased the expression of cleaved-caspase-3 in HCT116 cells (**Figure S2A**).

**Figure 5:**
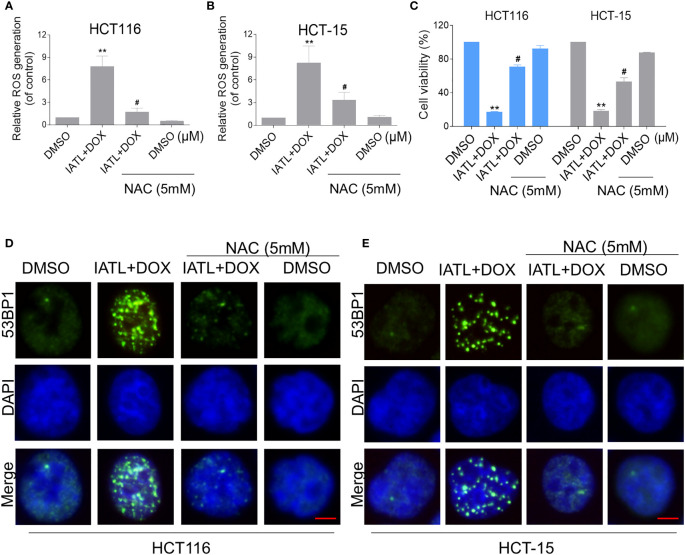
The combined treatment-induced accumulation of ROS and nuclear 53BP1 foci was significantly attenuated by NAC pretreatment in HCT116 and HCT-15 cells. **(A, B)** Cells were pretreated with NAC (5 mM) for 1 h before exposure to IATL and DOX. Intracellular ROS levels were measured after treated with IATL (20 µM) and DOX (1 µM) for 1 h. **(C)** Cells were pretreated with NAC (5 mM) for 1 h and cell viability was measured after treated with the combination for 24 h. **(D, E)** Cells were pretreated with NAC (5 mM) for 1 h and then treated with IATL (20 µM) and DOX (1 µM) for 20 h, the nuclear foci formation of 53BPl was detected. Scale bar = 5 µm. Data from three technical replicates (**p < 0.01 vs DMSO; ^#^p < 0.05 vs IATL+DOX).

We further explored the relationship between ROS production and the combined treatment-induced cell death. As shown in **Figures 5A–C**, the combined treatment-induced colon cancer cell death was rescued by pretreatment with the NAC. In addition, the combination treatment-induced increase in cleaved-caspase-3 expression was significantly reversed by NAC pretreatment (**Figure S2B**). Furthermore, the combined treatment-induced accumulation of nuclear 53BP1 foci was significantly attenuated by NAC pretreatment in HCT116 and HCT-15 cells, suggesting that DNA damage happened downstream of ROS production (**Figures 5D, E**).

### ROS-Mediated JNK Signaling Pathway Contributes to IATL-Induced Colon Cancer Cell Death

**Figure 6:**
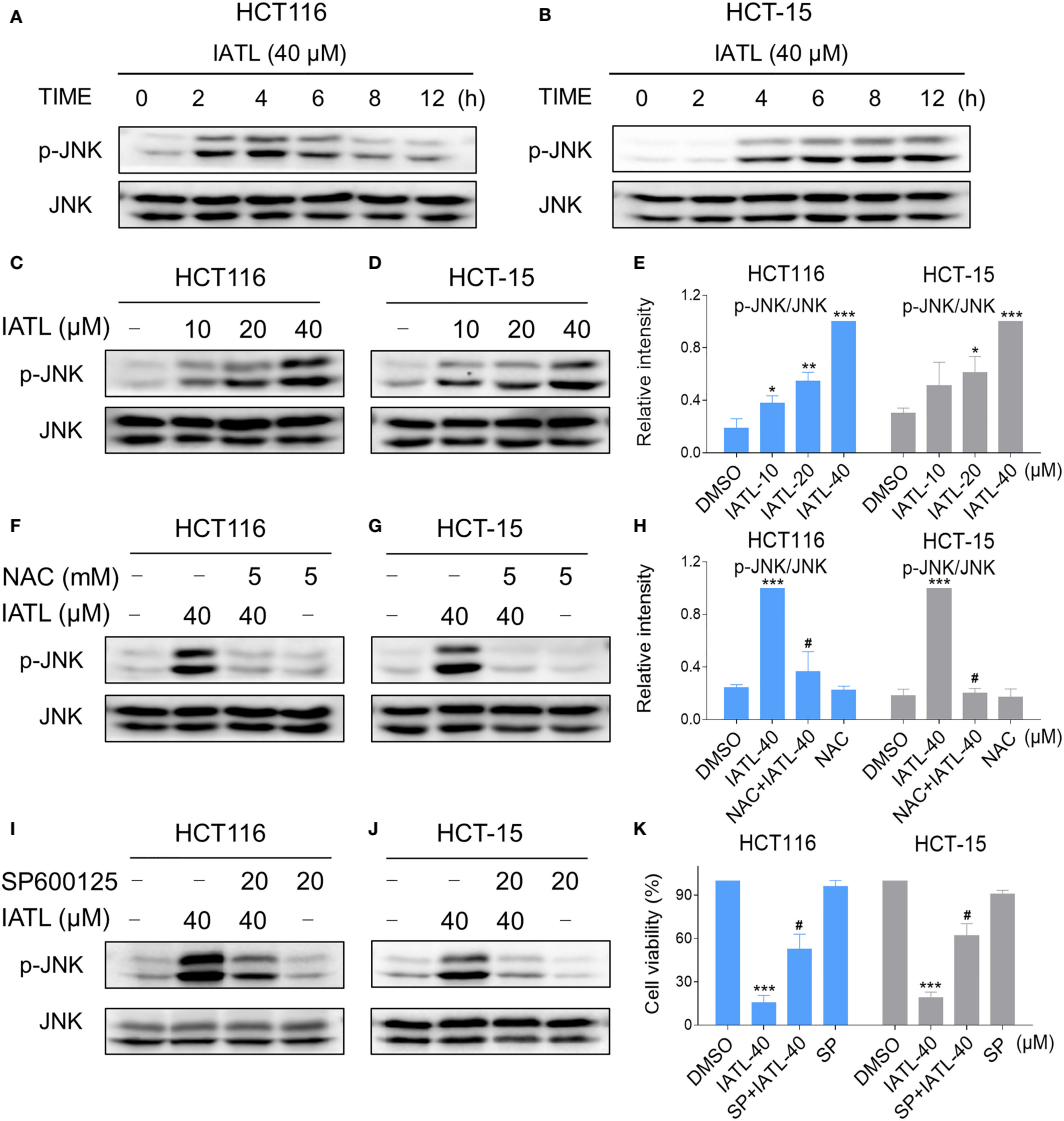
The JNK signaling pathway was activated in colon cancer cells after treated with IATL. **(A, B)** Phospho-JNK (p-JNK) and the total protein expression (JNK) in HCT116 **(A)**, and HCT-15 **(B)** cells upon IATL treatment was examined by western blot analysis. **(C–E)** HCT116 and HCT-15 cells were treated with various dosages of IATL for 6 h, then the expression of p-JNK and JNK were examined by western blot analysis. **(F–H)** Cells were pretreated with NAC (5 mM) for 1 h and then treated with IATL (40 µM) for 6 h, the expression of p-JNK and JNK were examined by western blot analysis. **(I, J)** Cells were pretreated with SP600125 (20 µM) for 1 h and then treated with IATL (40 µM) for 6 h, the expression of p-JNK and JNK were examined by western blot analysis. **(K)** Cells were pretreated with SP600125 (20 µM) for 1 h and cell viability was measured after treated with IATL (40 µM) for 24 h. Data from three technical replicates (*p < 0.05, **p < 0.01, ***p < 0.001 vs DMSO; ^#^p < 0.05 vs IATL-40).

Recent studies have shown that JNK signaling can be activated in response to various stimuli, including oxidative stress, apoptosis, irradiation and cytokines (21, 22). Here, we set out to verify whether the JNK signaling pathway was activated in colon cancer cells after treated with IATL. IATL treatment triggered the activation of JNK signaling pathway in a time-dependent (**Figures 6A, B**) and dose-dependent (**Figures 6C–E**) manner. Then we studied the connection between ROS generation and JNK activation in these cells. Since 40 µM IATL strongly induced p-JNK expression, we selected 40 µM IATL to analyze the ROS-mediated JNK signaling pathway. As expected, pretreatment with NAC protected against IATL-induced the activation of JNK signaling pathway (**Figures 6F–H**). To determine whether the activation of JNK signaling pathway was pivotal for the IATL-induced cell death, we used the specific JNK inhibitor SP600125 to inhibit the phosphorylation of JNK. As shown in **Figures 6I, J**, the phosphorylation of JNK induced by IATL was markedly attenuated by SP600125 pretreatment. Indeed, we found that the cell death induced by IATL was also significantly reversed when pre-treated with SP600125 (**Figure 6K**). Taken together, these results suggest that the ROS-mediated JNK activation is essential for the IATL-induced cell death in HCT116 and HCT-15 cells.

### IATL and DOX Cooperated to Activate ROS-Mediated JNK Signaling Pathway

**Figure 7:**
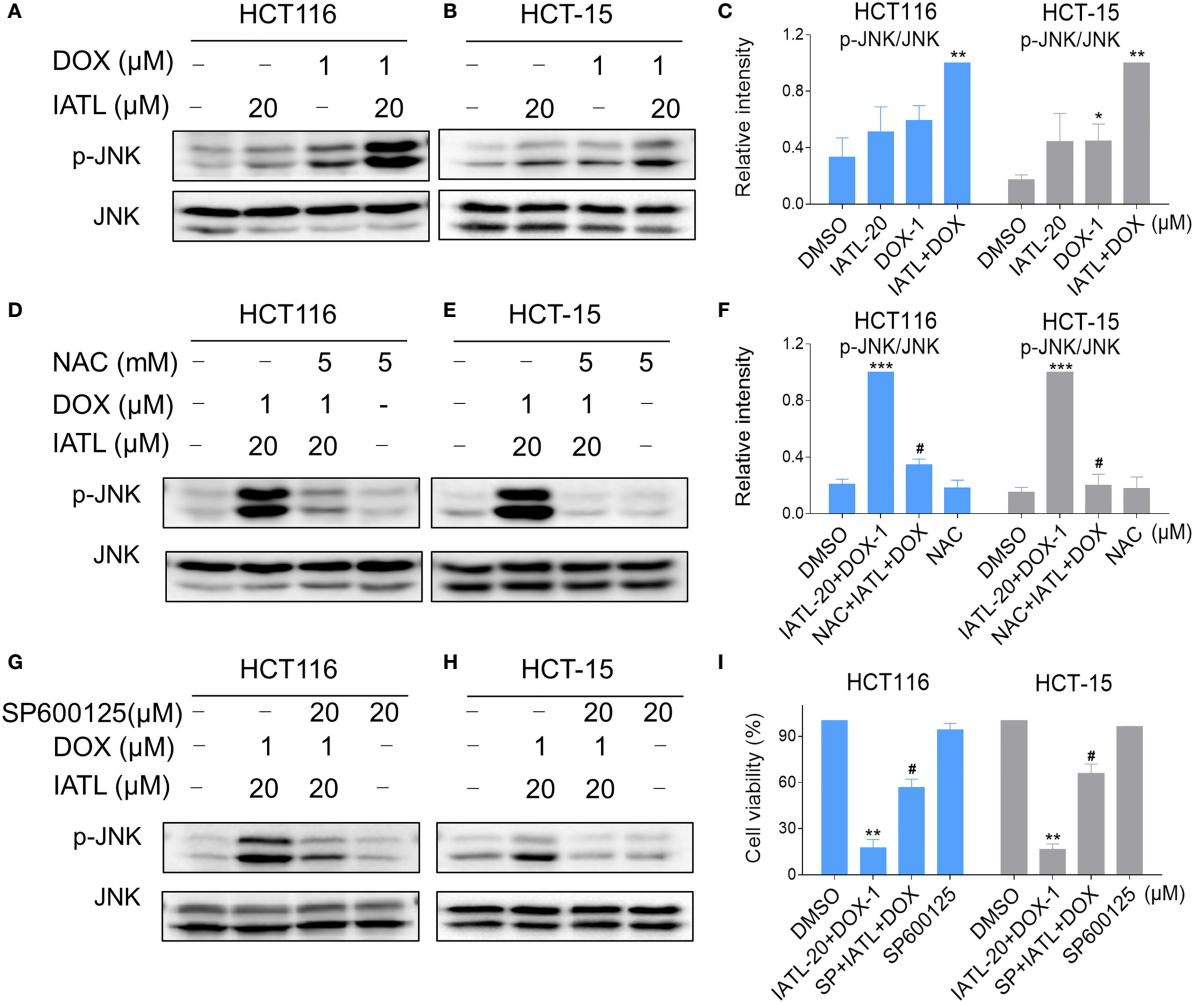
IATL and DOX cooperated to activate ROS-mediated JNK signaling pathway. **(A–C)** HCT116 and HCT-15 cells were treated with IATL (20 µM) or DOX (1 µM) alone or their cotreatment for 4 h, then the expression of p-JNK and JNK were examined by western blot analysis. **(D–F)** Cells were pretreated with NAC (5 mM) for 1 h and then treated with IATL (20 µM) and DOX (1 µM) for 4 h, the expression of p-JNK and JNK were examined by western blot analysis. **(G–H)** Cells were pretreated with SP600125 (20 µM) for 1 h and then treated with IATL (20 µM) and DOX (1 µM) for 4 h, the expression of p-JNK and JNK were examined by western blot analysis. **(I)** Cells were pretreated with SP600125 (20 µM) for 1 h, the cell viability was measured after treated with IATL (20 µM) and DOX (1 µM) for 24 h. Data from three technical replicates (*p < 0.05, **p < 0.01, ***p < 0.001 vs DMSO; ^#^p < 0.05 vs IATL-20+DOX-1).

Our results showed that IATL can activate JNK signaling pathway *via* ROS production. Subsequently, we investigated the connection between ROS generation and activation of JNK signaling pathway induced by the combined treatment in HCT116 and HCT-15 cells. Our results showed that the combination of 20 µM IATL and 1 µM DOX synergistically inhibited cell growth, induced ROS production, and promoted DNA damage. Thus, we used 20 µM IATL in combination with 1 µM DOX to investigate ROS-mediated JNK activation. As shown in **Figures 7A–C**, combined treatment resulted in greatly notable increases in JNK phosphorylation levels in both HCT116 and HCT-15 cells compared with IATL or DOX treatment alone. In addition, elevated phosphorylation of JNK was dramatically reversed after NAC pretreatment, suggesting that the activation of JNK signaling pathway is due to the intracellular ROS production in these cells (**Figures 7D–F**). Moreover, the effect of the combination treatment-induced cell death was reversed by SP600125 (**Figures 7G–I**). These results indicated that the activation of JNK signaling pathway is crucial for the synergistic effect of IATL and DOX.

## Discussion

**Figure 8:**
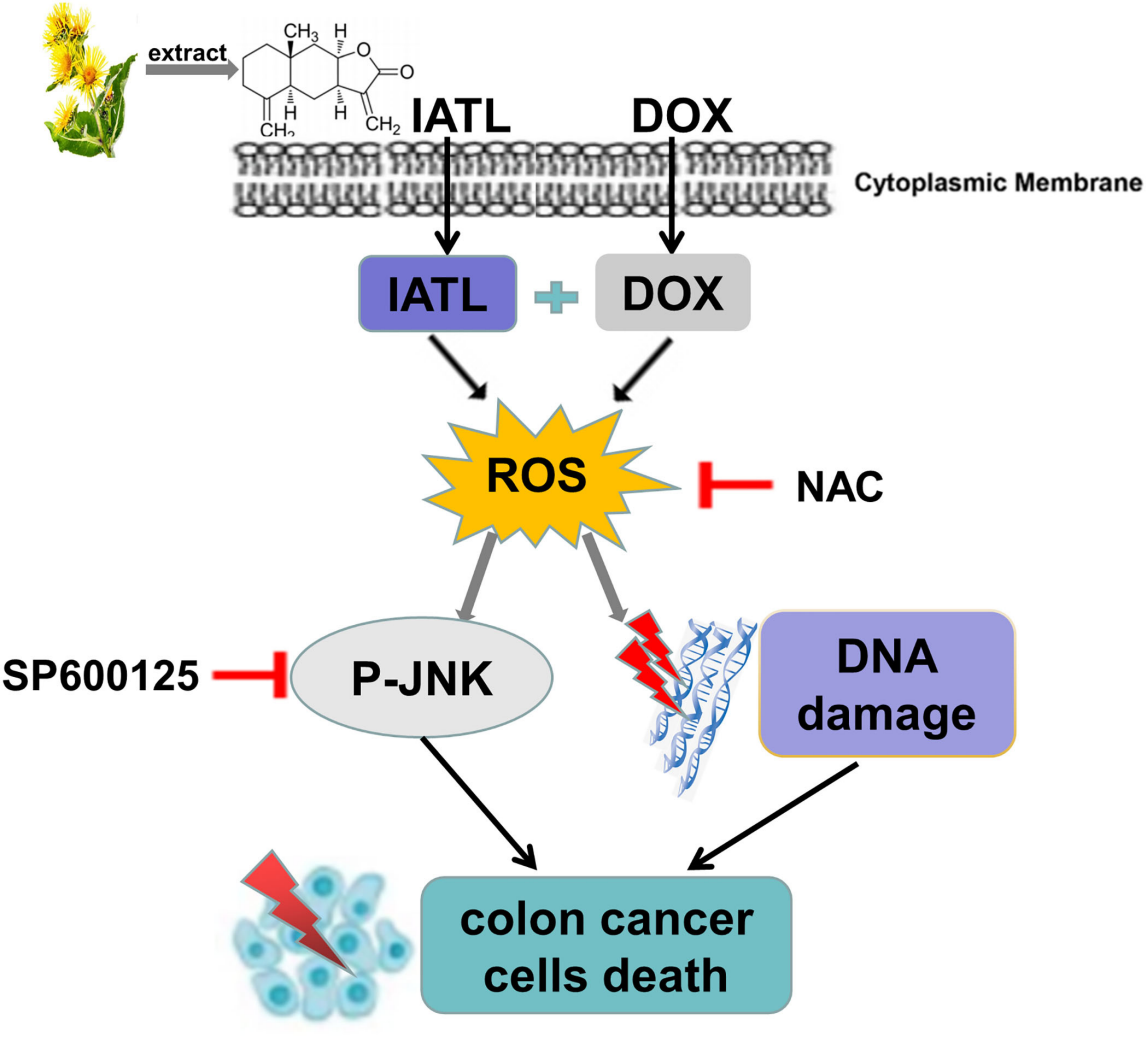
The proposed working model.

Previous studies have found that IATL has anti-cancer activity in a variety of cancer cells (23, 24). However, the role of IATL in colon cancer remains unclear. In the present study, we observed that IATL significantly inhibited colon cancer cell growth. Remarkably, we demonstrated that the combination of IATL and DOX synergistically inhibited colon cancer cell growth by activating ROS-mediated DNA damage and JNK signaling pathways (**Figure 8**). Thus, combined treatment with IATL and DOX may be a promising therapeutic strategy for colon cancer.

High expression of ROS makes cancer cells more sensitive to drugs that elevating ROS. Thus, increasing the ROS level to induce cancer cell death is a promising anti-cancer strategy (25, 26). In this study, we found that IATL combined with DOX markedly increased ROS levels in HCT116 and HCT-15 cells. Moreover, the cell death induced by the combination treatment was significantly reversed by NAC pretreatment, suggesting that ROS accumulation plays an important role in the synergistic action of IATL and DOX. These findings further support the notion that increasing ROS generation is a promising therapeutic strategy for cancer treatment.

ROS induces DNA damage through oxidation of nucleobases. 53BP1 is an indicator of DNA damage, and accumulated 53BP1 in the cell nucleus indicates the presence of DNA damage (27, 28). In our research, we found that ROS accumulation and subsequent DNA damage are central regulators of cell death. Combination of IATL and DOX significantly increased ROS accumulation and DNA damage in colon cancer cells compared with IATL or DOX alone. Importantly, NAC pretreatment reversed the combined treatment induced DNA damage in colon cancer cells.

JNK signaling pathway plays a crucial role in cell proliferation, cell differentiation, apoptosis and oxidative stress (21). Western blotting analysis suggested that JNK signaling pathway was activated in colon cancer cells following treatment with IATL. Moreover, IATL combined with DOX significantly enhanced the activation of JNK signaling pathway. Importantly, the JNK phosphorylation was abrogated by NAC in colon cancer cells, indicating that JNK signaling pathway is a downstream target of ROS.

In summary, our research demonstrated that IATL promoted the accumulation of intracellular ROS that resulted in DNA damage in colon cancer cells. In addition, we confirmed that IATL combined with DOX synergistically induced colon cancer cells death through ROS production and DNA damage. Our data suggests that the combination of IATL and DOX can be used as a new strategy for treatment of colon cancer.

## Data Availability Statement

The original contributions presented in the study are included in the article/**Supplementary Material**. Further inquiries can be directed to the corresponding authors.

## Author Contributions

CX and RC designed the study and wrote the manuscript. FW, RS, and PSZ performed the experiments. TZ, CQ, HS, SL, LJ, HP, XJ, and PZ collected and analyzed the data. All authors contributed to the article and approved the submitted version.

## Funding

Financial support from the Zhejiang Provincial Natural Science Foundation (LY20H310004, LZ22H160006), National College Students Innovation and Entrepreneurship Training Program (201910343025), National Natural Science Foundation of China (81672305), Wenzhou Municipal Science and Technology Bureau (Y2020733), and the Fundamental Research Funds for the Wenzhou Medical University (KYYW201929) are gratefully acknowledged.

## Conflict of Interest

The authors declare that the research was conducted in the absence of any commercial or financial relationships that could be construed as a potential conflict of interest.

## Publisher’s Note

All claims expressed in this article are solely those of the authors and do not necessarily represent those of their affiliated organizations, or those of the publisher, the editors and the reviewers. Any product that may be evaluated in this article, or claim that may be made by its manufacturer, is not guaranteed or endorsed by the publisher.
