# Supplementary material for: Isoalantolactone Enhances the Antitumor Activity of Doxorubicin by Inducing Reactive Oxygen Species and DNA Damage
Source: Front Oncol. 2022 Jan 25;12:813854. doi: 10.3389/fonc.2022.813854 (PMC8821528; doi:10.3389/fonc.2022.813854)
Supplement: Supplementary file 1 [file DataSheet_1.pdf]

**Supplementary data**

Figure S1

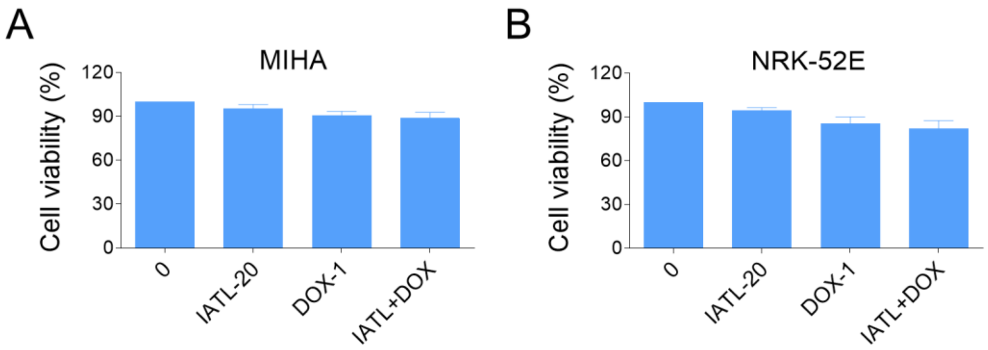


**Figure S1.** (A-B) Cell viability was measured after treated with IATL or DOX alone or their combination (20 μM IATL and 1 μM DOX) for 24 h.

Figure S2

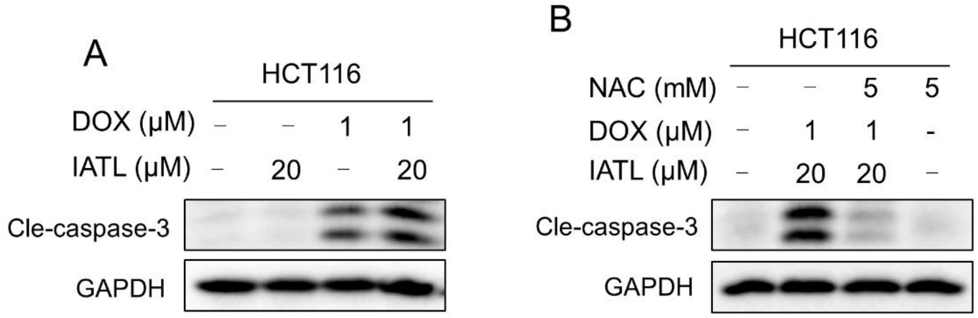


**Figure S2.** (A) HCT116 cells were treated with IATL or DOX alone or their combination for 20 h, the expression of cle-caspase-3 and GAPDH were examined by western blot analysis. (B) HCT116 cells were pretreated with NAC (5 mM) for 1 h and cell lysates were blotted with the indicated antibodies after treated with IATL and DOX combination for 20 h.
